# 

**Published:** 1918-03-02

**Authors:** 


RATES OF SUBSCRIPTION: Twelve months, 6/6; Six months, 4/-; three months, 2/-, post free to any address in the United Kingdom. Abroad, Twelve months, 8/8; Six months, 5/-; Three months, 2/6, post free. **THE HOSPITAL "Index" to Volume LXII., April 1917 to September 1917, is now ready, price 2½d. per copy, post free.** Address The Manager, THE HOSPITAL, "The Hospital" Building, 28/29 Southampton Street, Strand, London, W.C. 2.

*"LYSOL" EVANS*

*(Also known as "EVANSOL")*

*Neutral and Non-Corrosive.*

*In 4-oz., 8-oz., 16-oz. and 32-oz. Bottles.*

*Specify "LYSOL" EVANS and ensure a standard article that can be completely relied upon.*

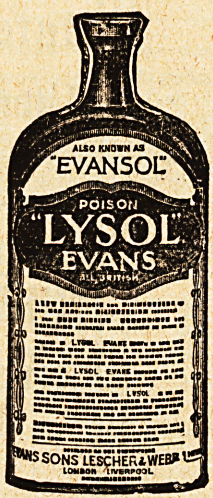

*Gives a Clear Solution.*

*In ½-gall and 1-gall Tins.*

*On application we will send Free a full 4-oz. Sample to Medical Men, Nurses and Institutions.*

*Evans Sons Lescher & Webb, Limited*

*60 Bartholomew Close, London.* *56 Hanover Street, Liverpool.*

### Matrons' and Sisters' Appointments.

Addenbrooke's Hospital, Cambridge.—Miss Charlotte D. Briscoe has been appointed assistant matron. She was trained at the Manchester Royal Infirmary and the General Infirmary, Bolton; has been sister at the City Hospital, Manchester, the Royal Sea-Bathing Hospital, Margate, and the 5th Southern General Hospital, T.F.N.S., Southsea; sister and night superintendent at the General Infirmary, Worcester, and assistant superintendent of the North Riding Nursing Association.

Bethnal Green Military Hospital, London, E.—Miss E. C. Robbins has been appointed sister. She was trained at the South Devon and East Cornwall Hospital, Plymouth, where she was afterwards night superintendent and surgical ward sister. She has also been sister-in-charge at the 4th Southern General Hospital, Plymouth.

Blackburn and East Lancashire Royal Infirmary.—Miss Olive Clemence has been appointed assistant matron of the above institution, at which she has been night sister.

Borough of Hornsey Isolation Hospital, Muswell Hill.—Miss Irene Webb has been appointed matron. She was trained at the London Hospital, and has been matron of the Fever Hospitals at Leicester, Bolton, and Bucknall, and of the Clare Hall Sanatorium, South Mimms, Barnet. She has also held the position of lady superintendent of the Women's Welfare Department, Daimler Motor Company, Coventry.

Cottage Hospital, Leek, Staffs.—Miss Mary Buchanan has been appointed nurse-matron. She was trained at the North Staffordshire Infirmary, where she has been sister-in-charge of various wards.

County Hospital, Taunton.—Miss Norah Portnall has been appointed night sister. She was trained at St. Bartholomew's Hospital, London, and has been sister at the Norfolk War Hospital, Thorpe, Norwich.

Durham County and Sunderland Eye Infirmary, Sunderland.—Miss E. G. Collingwood has been appointed sister. She was trained at the above-named institution and at the Royal Infirmary, Sheffield.

Ear and Throat Hospital, Birmingham.—Mrs. M. E. Burden has been appointed sister. She was trained at Leicester Royal Infirmary and Leicester Isolation Hospital, and has since been theatre sister at Brighton Ear and Throat Hospital, and at Loughborough General Hospital.

Essex County Hospital, Colchester.—Miss M. Flood has been appointed theatre sister. She was trained at Bury General Infirmary, where she was later night sister, and has been ward sister at Essex County Hospital, Colchester. Miss Florence Fox has been appointed home sister. She was trained at the above institution, has been ward sister there, and night maternity sister at Dundee Royal Infirmary. She has also done private nursing, and holds the C.M.B. certificate.

Red Cross Hospital, Perth.—Miss Annie Jameson has been appointed night sister. She was trained at Dundee Royal Infirmary, has been charge nurse at the Glasgow Eye Infirmary, has nursed in France with the Ulster Volunteer Force, and has been theatre sister at Croydon War Hospital and the Sutton Red Cross Hospital.

Royal Hospital for Sick Children, Edinburgh.—Miss J. Crawford has been appointed theatre sister. She was trained at King's College Hospital and at the Royal Hospital for Sick Children, Edinburgh.

Royal Infirmary, Liverpool.—Miss N. Gwynant Jones has been appointed sister. She was trained at St. Mary's Hospital, Paddington, and holds the certificate of the Central Midwives Board. She has since held the post of holiday sister and sister of the ante-natal and gynæcological wards at St. Mary's Hospital.

**NOTE.—Particulars of Appointments for insertion in this column will be welcomed.**

### A Happy Afternoon for Mental Defectives.

Mr. W. H. Walker, the Mental Deficiency Act Officer for the City of Hull, entertained a number of defectives (including some of the workhouse inmates) on Saturday, February 9. Councillor H. Dear, J.P., and Councillor F. Booth, J.P., attended, and spoke a few words in appreciation of the efforts of the promoter of the entertainment. This is the first time such an event has taken place in this particularly unfortunate class, comprising both adult and juvenile defectives, and it was a great success. A local gentleman, Mr. T. Hudson, trawler-owner, defrayed the cost and gave money to be distributed in coppers at the close. Mr. A. Proctor, of the Town Clerk's Department, took the chair.

### Recently Published Parliamentary Returns, etc.

Ministry of Reconstruction. Local Government Committee Report on Transfer of Functions of Poor-Law Authorities in England and Wales. Post free 4d.

General Principles Guiding the Treatment of Wounds. Post free 3d.

Public Health, England. Venereal Diseases (Anglesey, etc.) Order, December 5, 1917. Post free 1½d.

East India (Sanitary Measures). Report on Sanitary Measures in India in 1915-16. Vol. xlix. Cd. 8,873. Post free 1s. 8d.

Midwives (Ireland) Bill. Post free 3d.

National Health Insurance Bill. Post free 9d.

Ministry of Munitions. Health of the Munitions Worker. Post free 1s. 10d.

National Health Insurance. Medical Research Committee. Special Report Series No. 12. The Classification and Study of the Anserobic Bacteria of War Wounds. Post free 2s. 4d.

Report on the Administration of the National Relief Fund up to September 30, 1917. Post free 3d.

Public Health, England. Prevention of Epidemic, Endemic, or Infectious Diseases. Public Health (Notification of Infectious Diseases) Regulations, 1918, January 19, 1918. Post free 1½d.

Circular on same matter, January 19, 1918. Post free 1½d.

